# The serologically defined colon cancer antigen-3 (SDCCAG3) is involved in the regulation of ciliogenesis

**DOI:** 10.1038/srep35399

**Published:** 2016-10-21

**Authors:** Fangyan Yu, Shruti Sharma, Agnieszka Skowronek, Kai Sven Erdmann

**Affiliations:** 1 Department of Biomedical Science & Centre of Membrane Interactions and Dynamics, University of Sheffield, S10 2TN Sheffield, UK

## Abstract

A primary cilium is present on most eukaryotic cells and represents a specialized organelle dedicated to signal transduction and mechanosensing. Defects in cilia function are the cause for several human diseases called ciliopathies. The serologically defined colon cancer antigen-3 (SDCCAG3) is a recently described novel endosomal protein mainly localized at early and recycling endosomes and interacting with several components of membrane trafficking pathways. Here we describe localization of SDCCAG3 to the basal body of primary cilia. Furthermore, we demonstrate that decreased expression levels of SDCCAG3 correlate with decreased ciliary length and a reduced percentage of ciliated cells. We show that SDCCAG3 interacts with the intraflagellar transport protein 88 (IFT88), a crucial component of ciliogenesis and intraciliary transport. Mapping experiments revealed that the N-terminus of SDCCAG3 mediates this interaction by binding to a region within IFT88 comprising several tetratricopeptide (TRP) repeats. Finally, we demonstrate that SDCCAG3 is important for ciliary localization of the membrane protein Polycystin-2, a protein playing an important role in the formation of polycystic kidney disease, but not for Rab8 another ciliary protein. Together these data suggest a novel role for SDCCAG3 in ciliogenesis and in localization of cargo to primary cilia.

The cilium is a specialized membranous microtubule dependent protrusion of the plasma membrane dedicated to signal transduction as well as chemo- and mechanosensing^1^,^2^,^3^. Defects in cilia give rise to a number of human diseases so called ciliopathies like the Bardet-Biedel syndrome, the Joubert syndrome or the Meckel-Gruber syndrome, which are characterized by overall ciliary disruption resulting in retinal degeneration, mental retardation, skeletal abnormalities, or situs inversus^4^,^5^.

Cilia are formed by the transformation of the centrosome into a basal body localized in close proximity to the plasma membrane. In the case of primary (non-motile) cilia a 9 + 0 arrangement of microtubules is originating from the mother centriole of the basal body protruding the plasma membrane in an outward direction forming the so-called axoneme^6^,^7^. The proximal part of the cilium comprises the transition fibers, which connect the basal body with the periciliary membrane^8^,^9^. The transition fibers are followed more distally by the transition zone, a ciliary region believed to act as a molecular filter controlling transport of cargo in and out of the cilium^10^,^11^,^12^,^13^. There is no free diffusion of membrane proteins between the periciliary membrane and the ciliary membrane and entry and exit of membrane proteins is tightly controlled by a largely unknown mechanism. In contrast, soluble proteins appear to diffuse into the cilium lumen freely up to a certain molecular size, however proteins of a bigger size require specific transport processes into the cilium, which are related to nuclear transport processes^14^,^15^. In addition, cilia have a specialized ciliary protein translocation system for membrane and soluble proteins, the intraflagellar transport (IFT) system, which moves proteins into and within the cilium. This transport system consists of two main supramolecular IFT protein subcomplexes A and B, which are arranged in higher order protein arrays named IFT trains to move cargo along the axoneme microtubules via motor proteins^6^,^16^,^17^,^18^.

Currently, it is largely unclear how membrane proteins are entering the cilium and mechanisms how cargo is transported to the cilium are just emerging. However, membrane transport processes seem to be key for cilia formation and maintenance. An early step in ciliogenesis is the accumulation of Rab8 exchange factor Rabin8 at centrosomes^19^. Rabin8 is associated with the Bardet-Biedel-Syndrome (BBS) protein 1, a component of the BBSome which is formed by a core complex of seven BBS proteins and which plays a major role in delivering cargo to the cilium^20^. Rabin8 also leads to activation of the monomeric G-protein Rab8, which is important for cilia formation and cargo transport to cilia^20^. Accumulation of Rabin8 is Rab11 dependent trafficking processes and Rab11 is a major regulator of membrane recycling^19^,^21^. However, our understanding of the involvement of the recycling and endocytic pathway in ciliogenesis and cargo localization to cilia is incomplete and many molecular players are still elusive. Furthermore, the molecular cross talk between players of membrane trafficking and intraflagellar transport is largely unclear.

Here we focus on the role of the serologically defined colon cancer antigen-3 (SDCCAG3) in ciliogenesis. SDCCAG3 is a 45 kd protein consisting of a C-terminal coiled coil domain^22^. It has been shown before to localize to early and recycling endosomes and to interact with components of the retromer complex important for membrane receptor sorting^23^,^24^. Here we show that SDCCAG3 localizes also to primary cilia and interacts with the intraflagellar transport protein IFT88. Furthermore, we demonstrate that SDCCAG3 plays an important role in ciliogenesis and cargo localization in primary cilia.

## Results

### SDCCAG3 localizes to cilia

**Figure 1:**
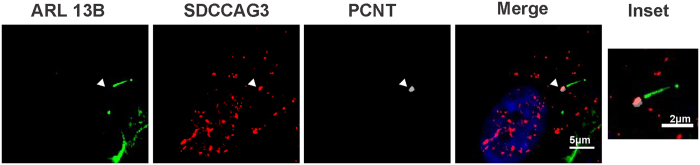
SDCCAG3 localizes to the cilia basal body. RPE1 cells stably expressing ARL13b-EGFP were serum-starved for 48 h to induce primary cilia, and then stained with anti-SDCCAG3 antibody (Ab2), anti-pericentrin antibody (PCNT), and DAPI. Images were obtained by fluorescence microscopy. Scale Bar represents 5 μm. Inset, scale bar represents 2 μm.

We have recently identified the serologically defined colon cancer antigen-3 (SDCCAG3) to be a novel endosomal protein^23^. Several proteins involved in endosomal membrane trafficking also play a role in ciliogenesis and cargo transport to cilia^25^,^26^,^27^,^28^. This prompted us to analyze a potential localization of SDCCAG3 to the primary cilium. Retinal pigment epithelial (RPE) cells stably expressing EGFP-Arl13b were stained for endogenous SDCCAG3 using a specific anti-SDCCAG3 antibody. Arl13b is a monomeric G-protein involved in membrane trafficking and is a well-established cilia marker protein^29^. Using the commercially available anti-SDCCAG3 antibody we observed localization of SDCCAG3 to the cilia basal body as revealed by double staining with pericentrin (PCNT), which serves as a basal body marker (Fig. 1). In RPE cells basal body localization of SDCCAG3 was observed in about 10% of ciliated cells. We observed similar basal body staining for SDCCAG3 also in mouse Inner medullary Connecting Duct (IMCD)−3 cells (in 29% of the cells) and in human embryonic kidney (HEK) cells (in 22% of the cells) (supplementary figure S2). We have validated the suitability of our anti-SDCCAG3 antibodies for immunofluorescence by performing corresponding siRNA experiments (supplementary figure S1).

### Amino acids 1-100 of SDCCAG3 are necessary and sufficient for basal body localization

**Figure 2:**
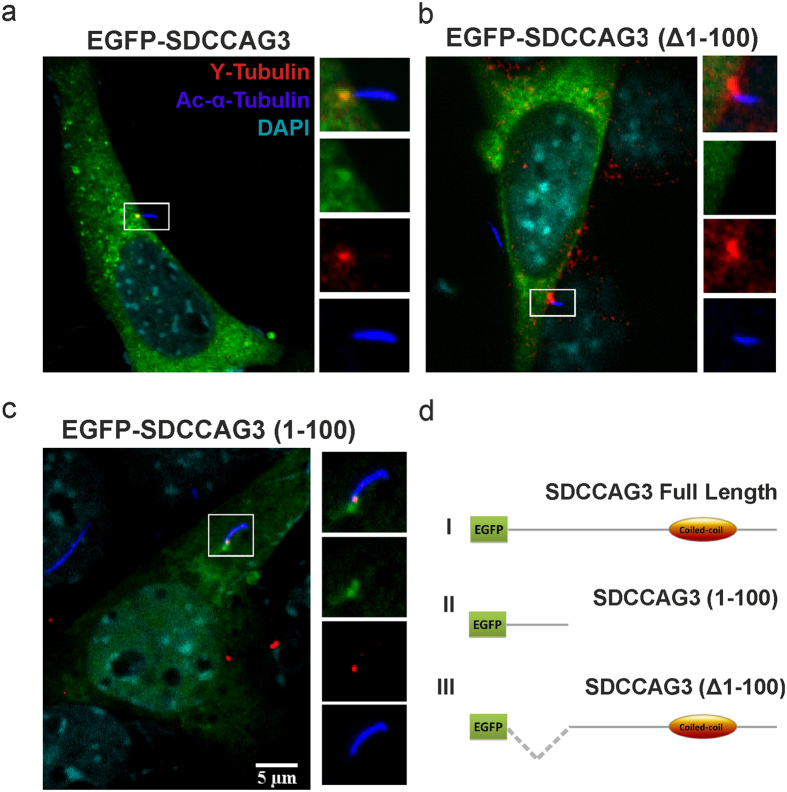
Localization of transfected EGFP-SDCCAG3 to cilia in IMCD3 cells. IMCD3 cells were transfected with the indicated EGFP-SDCCAG3 expression constructs (green) and stained for the cilia axoneme marker acetylated tubulin (blue) and the basal body marker gamma-tubulin (red). **(a)** Full-length EGFP-SDCCAG3 co-localized with gamma-tubulin at the basal body. **(b)** A deletion construct of SDCCAG3 lacking amino acids 1-100 (Δ1-100) did not co-localize with gamma-tubulin at the basal body. **(c)** A truncated version of SDCCAG3 comprising only amino acids 1-100 was sufficient for basal body localization of SDCCAG3. Images are representative of three different experiments. Scale bar represents 5μm. **(d)** A schematic diagram of the transfected EGFP-SDCCAG3 expression constructs showing the full length protein (I), the construct comprising only amino acids 1-100 (II) and SDCCAG3 lacking amino acids 1-100 (III).

Next we tested whether transfected EGFP-SDCCAG3 could also localize to cilia. IMCD3 cells were transfected with an expression construct for EGFP-SDCCAG3 and stained for endogenous acetylated tubulin (axoneme marker) as well as gamma-tubulin (basal body marker). We observed accumulation of EGFP-SDCCAG3 at basal bodies in 62% of the cells as indicated by co-localization with gamma-tubulin (Fig. 2a). This confirmed our results obtained using anti-SDCCAG3 antibodies that SDCCAG3 can localize to the cilia basal body of primary cilia.

In an attempt to learn more about the molecular requirements for SDCCAG3 localization to cilia we tested a truncated version of SDCCAG3 devoid of the first N-terminal 100 amino acids (Fig. 2d). Interestingly, this construct lost completely its ability to localize to cilia (Fig. 2b). In contrast a truncated version of SDCCAG3 comprising only amino acids 1-100 localized to the cilia basal body but similar to EGFP-SDCCAG3 (full-length) we could not observe this construct in the axoneme (Fig. 2c,d). In summary, we can conclude that the N-terminal amino acids 1-100 of SDCCAG3 are necessary and sufficient for basal body localization of SDCCAG3.

### Depletion of SDCCAG3 correlates with a reduced percentage of ciliated cells and with decreased cilia length

**Figure 3:**
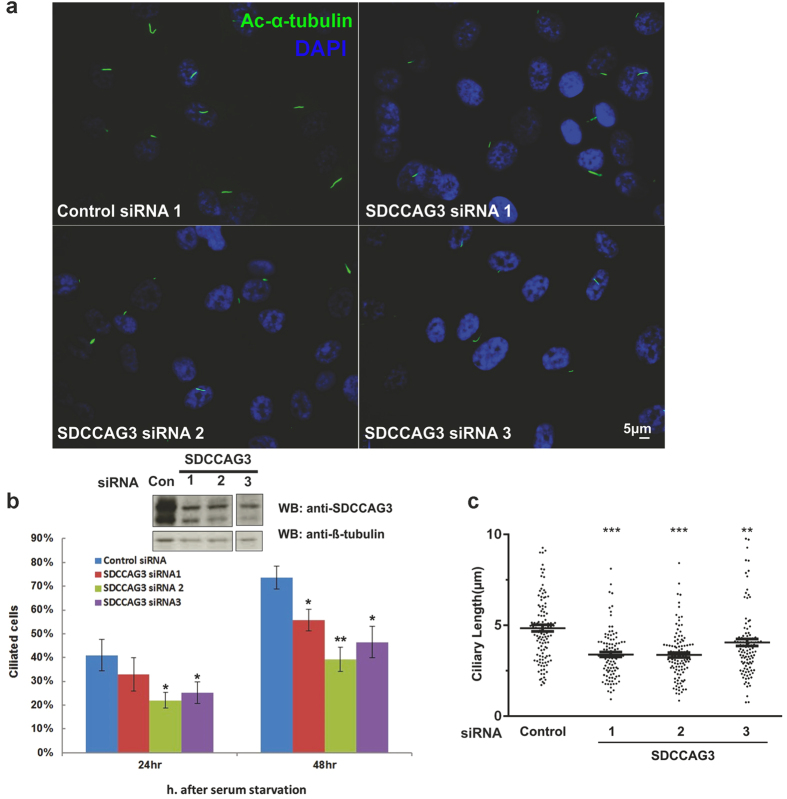
SDCCAG3 depletion impairs ciliogenesis. **(a)** Representative figures of ciliated RPE1 cells transfected with SDCCAG3 or control siRNAs. RPE1 cells were transfected with the indicated siRNA. After 24 h, cells were incubated in low serum for 48 h and immunostained with anti-acetylated-α-tubulin antibody. Scale bar represents 5 μm. **(b)** Upper panel, the knockdown efficiency of three different SDCCAG3 siRNAs is demonstrated by western-blotting. Cropped blots are shown, full-length blots are presented in supplementary figure S6. Lower panel, RPE1 cells were transfected with the indicated siRNA. 24 h post transfection, cells were starved for an additional 24 h or 48 h. Cilia were labelled with anti-acetylated-α-tubulin antibody and the percentages of ciliated cells were quantified. (n = the number of cilia, n > 300; N = number of independent experiments, N = 3; unpaired t-test). **(c)** The length of cilia at 48 hours post serum starvation was measured (n > 100, N = 3, F-test). *p < 0.05, **p < 0.01. ***p < 0.001. Error bars represent s.e.m.

In order to analyse whether SDCCAG3 might play a role in cilia formation we transfected RPE cells with siRNA targeting SDCCAG3. We applied three independent siRNAs to minimize analysing off-target effects and confirmed knockdown of SDCCAG3 by western-blotting (Fig. 3b). SDCCAG3 appears usually in multiple bands due to multiple phosphorylations as described before (23). We observed that reduced expression levels of SDCCAG3 strongly correlated with a reduced percentage of ciliated cells (Fig. 3a,b). In addition, we noticed that the overall cilia length was decreased in the case of SDCCAG3 depletion (Fig. 3c).

Cilia show a highly dynamic behaviour during cell cycle; to rule out that knockdown of SDCCAG3 has a major effect on the cell cycle of RPE cells and thus is indirectly affecting ciliogenesis, we transfected RPE cells with SDCCAG3 siRNA and determined the cell cycle status of individual cells using propidium iodide staining combined with flow cytometry analysis. We did not observe any changes in the relative distribution of different cell cycle stages comparing control or SDCCAG3 siRNA transfected cells (supplementary figure 3). Thus we conclude that the effect of siRNA mediated SDCCAG3 depletion on ciliogenesis and cilia length is not due to changes in the cell cycle distribution of RPE cells.

**Figure 4:**
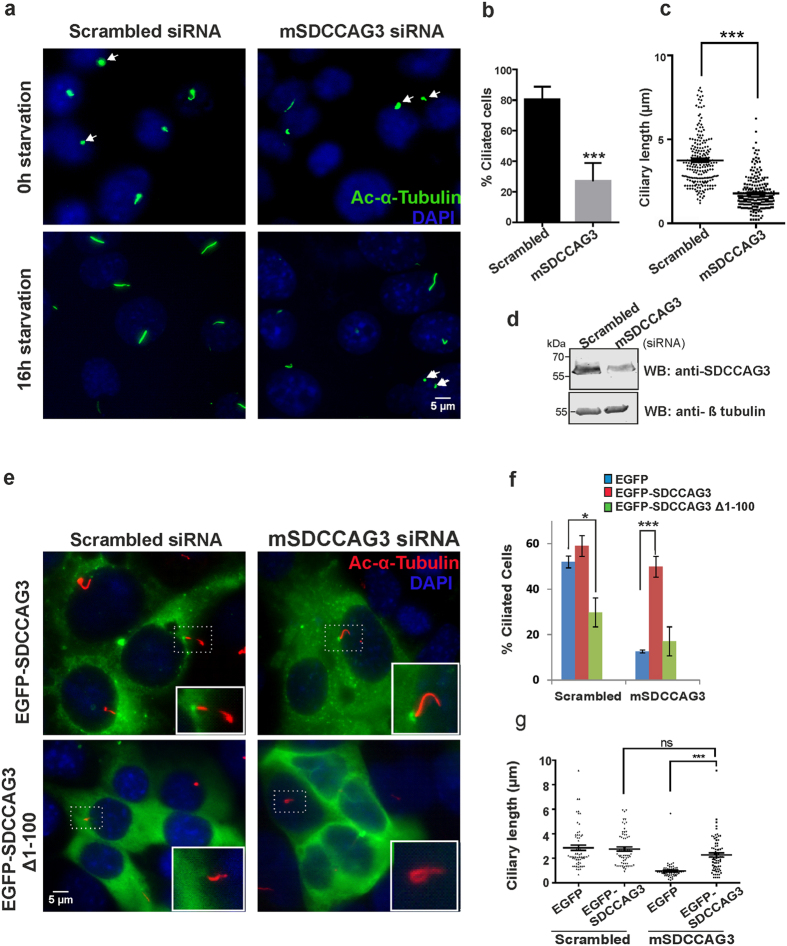
Impaired ciliogenesis observed after transfection of mouse IMCD3 cells with mSDCCAG3 siRNA can be rescued by overexpression of human SDCCAG3. **(a)** IMCD3 cells were transfected with scrambled or siRNA targeting mouse SDCCAG3 for 48 hours and then cultured without serum for 0 and 16h and stained with acetylated tubulin (green) or DAPI (blue). Arrows represent nascent cilia. Scale bar represents 5μm. **(b)** Quantification of the percentage of ciliated cells from three independent experiments; unpaired t-test, error bar represents ± s.e.m **P < 0.01. **(c)** Cells were treated as described in (a) and ciliary length was measured 16 hours post-serum starvation (N = 3, F-test,***p < 0.001). **(d)** Immunoblot analysis of knockdown of SDCCAG3 upon treatment with mSDCCAG3 siRNA. Cropped blots are shown, full-length blots are presented in supplementary figure S6. **(e)** Localization of transfected human EGFP-SDCCAG3 or EGFP-SDCCAG3 Δ1-100 in IMCD3 cells treated with scrambled or mSDCCAG3 siRNA and stained with acetylated tubulin (red) and DAPI (blue). Scale bar represents 5μm. **(f)** Quantification of the percentage of ciliated cells showing rescue of the ciliogenesis defect in mSDCCAG3 siRNA treated cells upon expression of full-length EGFP-SDCCAG3 but not upon EGFP alone or EGFP-SDCCAG3 Δ1-100 expression. N = 3, unpaired t-test,*p < 0.05, ***p < 0.001, error bars represent ± s.e.m. (**g**) Quantification of ciliary length in scrambled or mSDCCAG3 siRNA treated cells after 16 hours of serum-starvation and upon expression of full-length EGFP-SDCCAG3 or upon expression of EGFP alone. (N = 3, F-test, ***p < 0.001, ns-not significant).

Next we tested whether the observed phenotype upon SDCCAG3 depletion could be rescued by re-expression of SDCCAG3. Similar to human RPE cells mouse IMCD3 cells showed a reduced percentage of ciliated cells after siRNA mediated SDCCAG3 depletion (Fig. 4a,b). Furthermore, we also observed a reduction in ciliary length upon SDCCAG3 knockdown (Fig. 4c,d). To rescue this phenotype we transfected IMCD3 cells with an expression construct for human SDCCAG3, which is resistant to the applied siRNA that targets only the endogenous mouse SDCCAG3. Expression of EGFP-SDCCAG3 in SDCCAG3 siRNA treated cells led to a complete rescue of the phenotype restoring the percentage of ciliated cells to almost control levels (Fig. 4e,f). In contrast expression of the EGFP-Δ1-100-SDCCAG3 construct was not able to rescue the phenotype. Interestingly, expression of EGFP-SDCCAG3Δ1-100 led to a reduced number of cilia also under control conditions (control siRNA), suggesting that this construct exerts a dominant negative effect interfering with endogenous SDCCAG3 function. Finally, we could also rescue the ciliary length in SDCCAG3 depleted cells upon expression of EGFP-SDCCAG3 but not upon expression of EGFP (Fig. 4g).

### The serologically defined colon-cancer antigen 3 interacts with the intraflagellar transport protein 88

Above we have demonstrated that amino acids 1-100 are crucial for SDCCAG3 localization to cilia and also for SDCCAG3´s ability to rescue numbers of ciliated cells in SDCCAG3 knockdown cells. In order to identify potential interacting proteins for this region we applied the yeast two-hybrid system. Using the first N-terminal 100 amino acids of SDCCAG3 as bait, we identified the intraflagellar transport protein-88 (IFT88) as a potential binding partner. IFT88 is well known for its role in ciliogenesis and cilia cargo transport and thus was a promising candidate for a functionally relevant novel SDCCAG3 binding protein^30^,^31^.

**Figure 5:**
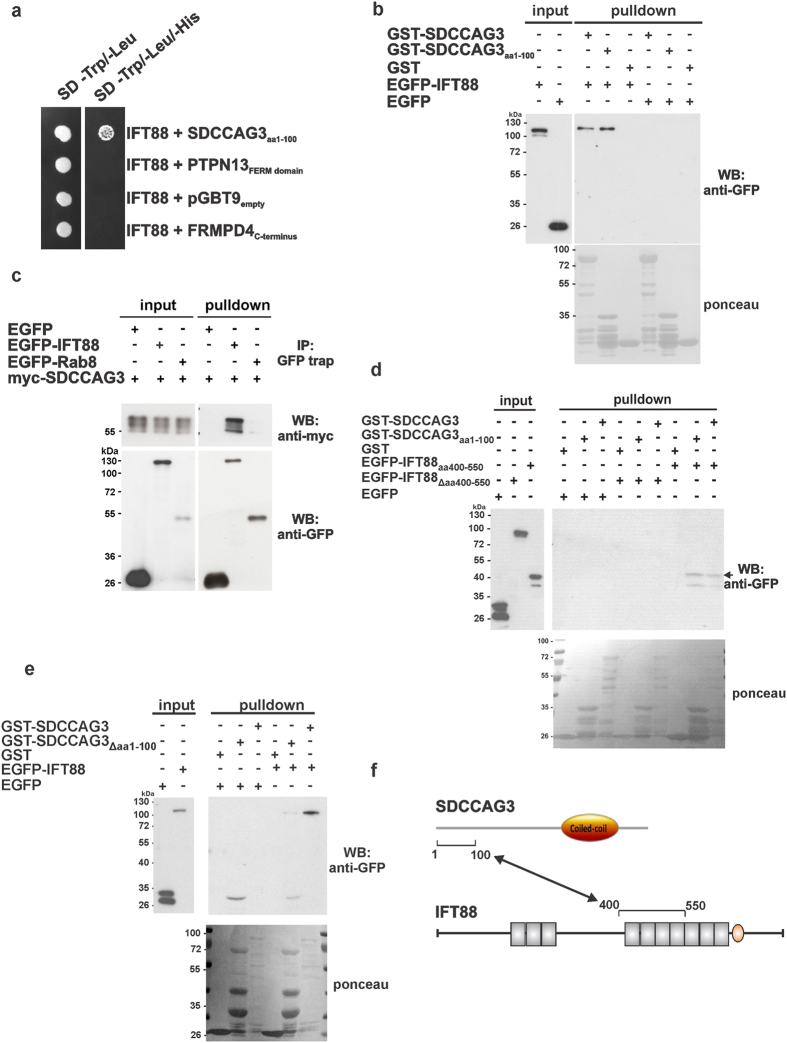
SDCCAG3 interacts with the intraflagellar transport protein IFT88. **(a)** Yeast two-hybrid analysis of the interaction between amino acids 1-100 of SDCCAG3 with IFT88. An expression construct for IFT88 (full-length) was co-transformed into yeast cells with expression vectors for SDCCAG3 (amino acids 1-100), or the indicated negative control plasmids (FRMPD4 = Ferm and PDZ domain containing protein 4, PTPN13 = Protein typrosine phosphatase 13, pGBT9 = empty bait expression vector). Growth on media deficient for tryptophan (Trp), leucine (Leu) and histidine (His) indicates interaction of the corresponding proteins. **(b)** GST-SDCCAG3 interacts with full-length IFT88. Lysate of Cos7 cells transiently transfected with EGFP or EGFP-IFT88 expression constructs were incubated with purified GST, GST-SDCCAG3 and GST-SDCCAG3 1-100aa beads respectively. The pulldown result was analyzed by western blotting using an anti-GFP antibody. **(c)** GFP-trap pulldown of EGFP-IFT88 and Myc-SDCCAG3. Cos7 cells were transiently transfected with expression plasmids for myc-SDCCAG3 or the indicated GFP fusion proteins. Corresponding cell lysates were mixed as indicated, and subsequently subjected to GFP-Trap^®^_A pulldown. Bound proteins were detected via western blot with anti-myc and anti-GFP antibody. Cropped blots are shown, full-length blots are presented in supplementary figure S6. **(d)** Amino acids 400-550 of IFT88 are sufficient and necessary for interaction with SDCCAG3. HEK293 cells were transfected with expression constructs for EGFP, EGFP-IFT88 aa400-550 or EGFP-IFT88Δaa400-550. After 48 h, cell lysates were incubated with purified GST, GST-SDCCAG3 1-100aa or GST-SDCCAG3 glutathione agarose beads; protein complexes were separated by SDS–PAGE and detected with anti-GFP antibody. The arrow indicated the position of EGFP-IFT88 aa400-550. **(e)** Amino acids 1-100 of SDCCAG3 are required for interaction with IFT88. The indicated SDCCCAG3 GST-fusion proteins were incubated with lysate of EGFP or EGFP-IFT88 transfected HeLa cells. Bound proteins were separated by SDS-PAGE and detected with anti-GFP antibody. **(f)** Schematic diagram of the interaction of SDCCAG3 with IFT88. Ovals indicate coiled coil domains; the grey rectangles represent tetratricopeptide repeats (TPR).

Interaction of IFT88 with SDCCAG3 is specific based on yeast two-hybrid criteria as no interaction could be detected with a number of control proteins like the FERM domain of the protein tyrosine phosphatase PTPN13 or the FERM and PDZ domain containing protein 4 (FRMPD4) (Fig. 5a). In order to validate this interaction independently from the yeast two-hybrid system we performed glutathione-S-transferase (GST)-pulldown experiments using purified recombinant GST-SDCCAG3 proteins and lysate of EGFP-IFT88 overexpressing HeLa cells. Interaction of EGFP-IFT88 could be observed with either full-length SDCCAG3 or with the truncated SDCCAG3 version comprising amino acids 1-100 only but not with GST alone (Fig. 5b). Next we tested whether this interaction can be detected by a reverse approach by pulling down IFT88. HeLa cells were transfected with expression constructs for Myc-SDCCAG3 and EGFP-IFT88 and a GFP pulldown assay was performed. Myc-SDCCAG3 co-precipitated with EGFP-IFT88 but not with EGFP alone or EGFP-Rab8, which were used as controls, indicating specificity of the interaction (Fig. 5c). In summary our yeast two-hybrid results together with our GST- and GFP-pulldown experiments indicate that SDCCAG3 is capable of interacting with IFT88.

### Mapping of the IFT88/SDCCAG3 binding site

To identify the binding region of SDCCAG3 within IFT88 we performed a series of mapping experiments using the yeast two-hybrid system and GST-pulldown experiments. First, we mapped the binding site to be within amino acids 400 to 550 of IFT88 using the yeast two-hybrid system (supplementary figure S4). Furthermore, we could show that amino acids 400 to 550 are sufficient for interaction of IFT88 with SDCCAG3, because a GFP fusion protein comprising only these amino acids still interacts with GST-SDCCAG3 or GST-SDCCAG3 (aa 1-100) as confirmed by pulldown experiments (Fig. 5d). We also showed that a deletion construct of IFT88 devoid of amino acids 400–550 is not able to interact with SDCCAG3 (Fig. 5d), suggesting that amino acids 400 to 550 of IFT88 are not only sufficient but also necessary for the interaction of IFT88 with SDCCAG3. Interestingly, amino acids 400–550 comprise several tetratricopeptide repeats (TPR). The TPRs represent a 34 amino acid long motif arranged in two alpha-helices often found in tandem arrays and known to mediate protein/protein interaction^32^. Finally, we tested whether amino acids 1-100 of SDCCAG3 are necessary for interaction with IFT88. We could show that binding of IFT88 to a GST-fusion protein of SDCCAG3 lacking amino acids 1-100 was greatly reduced compared to full-length SDCCAG3 (Fig. 5e). We have noticed that the coiled coil domain of SDCCAG3 is capable of multimerization (data not shown). Thus the weak IFT88 binding we observed with the SDCCAG3Δ1-100 construct could be due to multimerization of SDCCAG3Δ1-100 with endogenous full-length SDCCAG3. However, we cannot exclude a very weak second binding site in SDCCAG3 for IFT88. In summary we have mapped the interaction of SDCCAG3 and IFT88 to take place between amino acids 1-100 of SDCCAG3 and amino acids 400–550 of IFT88 (Fig. 5f).

### SDCCAG3 and IFT88 both localize at centrosomes and cilia

**Figure 6:**
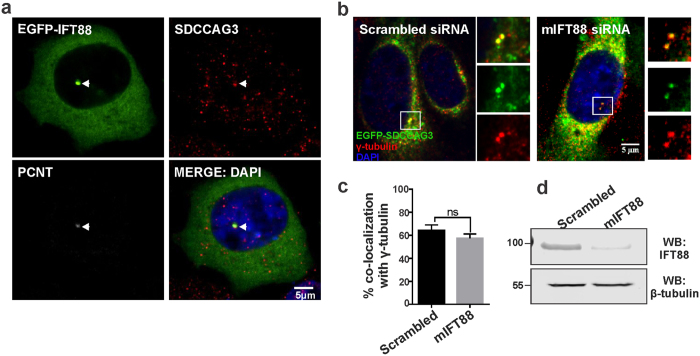
Co-localization of SDCCAG3 and IFT88 in cells. **(a)** Co-localization of SDCCAG3 and EGFP-IFT88 at centrosome. HeLa cells were grown on coverslip and transiently transfected with an expression construct for EGFP-IFT88. After 20 hours, cells were fixed and immunostained for SDCCAG3 and pericentrin (PCNT). Arrows indicate the co-localization at centrosomes. Scale bar represents 5 μm. **(b)** Localization of SDCCAG3 to the basal body is independent of IFT88. IMCD3 cells treated with scrambled or mouse IFT88 siRNA and transfected with EGFP-SDCCAG3 were stained with gamma-tubulin antibody. Scale bar represents 5 μm. **(c)** Quantification of co-localization of EGFP-SDCCAG3 with gamma-tubulin in cells treated with the indicated siRNA; n = 3, unpaired t-test, error bars ± s.e.m, ns = non-significant. **(d)** Immunoblot analysis validating knockdown of IFT88 in mIFT88 siRNA treated IMCD3 cells. Cropped blots are shown, full-length blots are presented in supplementary figure S6.

Next we analyzed whether SDCCAG3 and IFT88 co-localize in cells. Previously, we have described a centrosomal localization of SDCCAG3 in HCT116 cells and IFT88 has been described to localize at centrosomes before^23^,^33^. Thus we analyzed a potential co-localization of both proteins at centrosomes using HeLa cells and immunofluorescence analysis. We were prevented from performing double immunofluorescence analysis for SDCCAG3 and IFT88 directly because our antibodies to both proteins were derived from rabbits. Thus we analyzed co-localization of endogenous SDCCAG3 with transfected EGFP-IFT88. Both proteins were co-localizing at centrosomes as demonstrated by the presence of the centrosomal marker protein pericentrin (PCNT) (Fig. 6a). In order to analyze whether IFT88 is important for centrosome localization of SDCCAG3, we transfected IMCD3 cells with siRNA targeting IFT88 leading to IFT88 depletion (Fig. 6d). However, SDCCAG3 was still efficiently recruited to the centrosome as demonstrated by co-localization with gamma-tubulin (Fig. 6b,c). Thus we concluded that IFT88 is not necessary for SDCCAG3 recruitment to centrosomes, however we cannot rule out that the residual amount of IFT88 upon knockdown prevents us from observing a phenotype.

Next we investigated localization of IFT88 in ciliated RPE cells stably expressing Arl13b. We observed co-localization of endogenous IFT88 with EGFP-Arl13b in the cilium and localization of IFT88 to a region proximal to the basal body (transition zone) as described before (supplementary figure S5)^34^,^35^. We noticed also a partial overlap with the cilia basal body marker pericentrin. As described above, SDCCAG3 also localizes to the basal body of primary cilia in RPE cells suggesting a partial overlap between SDCCAG3 and IFT88 localization also in cilia.

### Depletion of SDCCAG3 impairs ciliary localization of Polycistin-2

**Figure 7:**
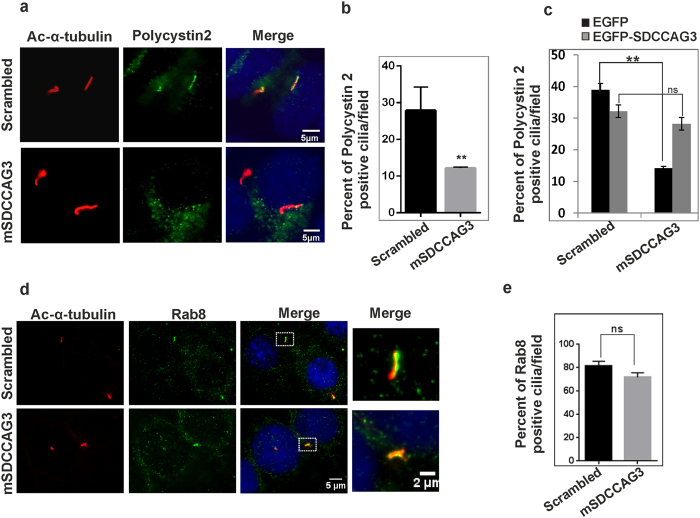
SDCCAG3 depletion impairs Polycystin-2 localization to primary cilia. **(a)** IMCD3 cells transfected with control siRNA or mSDCCAG3 siRNA for 48 h were serum starved overnight (16 hours). Cells were then fixed and immunostained with anti-acetylated-α-tubulin antibody (red), anti-polycystin-2 (green) antibody and DAPI (blue). Scale bar represents 5 μm. **(b)** Quantification of Polycystin-2 localization to cilia after SDCCAG3 knockdown. The number of Polycystin-2 positive cilia of siRNA transfected cells were quantified. **(c)** Rescue of ciliary Polycystin-2 upon overexpression of human EGFP-SDCCAG3 in mSDCCAG3 siRNA treated IMCD3 cells. IMCD3 cells were transfected with siRNA targeting mouse SDCCAG3, after 24 hours cells were also transfected with expression plasmids for EGFP or EGFP-SDCCAG3 for another 48 hours. Transfected cells were subsequently serum starved for 16 hours and stained with anti-acetylated-α-tubulin (blue) and anti-Polycystin-2 (red) antibodies. The number of cilia positive for Polycystin-2 was quantified as above; n = 3, unpaired t-test, **p < 0.05,error bars ± s.e.m. **(d)** Depletion of SDCCAG3 did not alter localization of Rab8 to cilia in IMCD3 cells transfected with mSDCCAG3 or scrambled siRNA. Cells were treated with the indicated siRNAs as described above and stained for Rab8, acetylated-α-tubulin and DAPI. Images were taken by confocal microscopy. Scale bar represents 5 μm. **(e)** Quantification of the percentage of Rab8 positive cilia in SDCCAG3 siRNA and control siRNA treated cells; n = 3, unpaired t-test, error bars ± s.e.m, ns = non-significant.

IFT88 is part of the inflagellar transport complex B important for cargo transport into and within the cilium. Having demonstrated that SDCCAG3 is interacting with IFT88 we tested whether depletion of SDCCAG3 also affects cargo localization to cilia. The transmembrane protein Polycystin-2 is an established ciliary cargo and it has been shown to form a complex with the exocyst protein Sec10 together with IFT88, which is required for its ciliary localization^36^,^37^,^38^. Given our finding that SDCCAG3 also interacts with IFT88 we tested whether SDCCAG3 could regulate Polycystin-2 localization to cilia. We transfected IMCD3 cells with siRNA targeting SDCCAG3 and monitored the cilia for the presence of Polycisitin-2 using immunofluorescence. We observed a significantly reduced localization of Polycystin-2 to cilia in SDCCAG3 siRNA treated cells compared to cells treated with control siRNA (Fig. 7a,b). Moreover, this effect could be rescued upon expression of an EGFP fusion protein with human SDCCAG3 suggesting that SDCCAG3 depletion is responsible for the observed lack of Polycystin-2 at cilia (Fig. 7c). In contrast, we did not observe any difference in Rab8 (another known ciliary protein) localization to cilia in SDCCAG3 depleted IMCD3 cells (Fig. 7d,e)^20^. Thus besides having an effect on the formation of cilia, SDCCAG3 also affects localization of selective cargo to cilia.

## Discussion

Here we show that the endosomal protein SDCCAG3 is important for cilia formation and cargo localization to cilia. Using antibodies recognizing endogenous SDCCAG3 and overexpression of EGFP-tagged SDCCAG3 we demonstrate that SDCCAG3 is a novel ciliary protein localizing to the basal body. The localization of SDCCAG3 to cilia was highly specific, as a truncated version of SDCCAG3 lacking the N-terminal 100 amino acids did not localize to cilia. Our findings are also in line with a recently performed large-scale proteomic approach to characterize the centrosome-cilium interface using proximity dependent biotinylation, where SDCCAG3 was identified in the proximity interactome of several centriolar proteins^39^.

Previous studies have shown that SDCCAG3 localizes to early and recycling endosomes and that it regulates cell surface expression of the tumor necrosis factor receptor, suggesting a role in membrane trafficking^22^,^23^. Moreover, SDCCAG3 forms a complex with other components regulating membrane trafficking like ARF6 GTPase activating protein GIT-1 or the retromer component VPS35 (vaculoar protein sorting associated protein 35)^23^,^24^. Thus SDCCAG3 appears to play a role in particular in the endocytic pathway likely regulating cargo transport or sorting.

There is good evidence that the endocytic pathway plays an important role in the formation and maintenance of primary cilia likely by delivering cargo-containing vesicles to the ciliary base or by participating in cargo sorting processes at stages before or at the cilium. In a screen for proteins regulating cilia length several proteins with established functions in the endocytic pathway have been identified^26^. Knockdown of the protein tyrosine phosphatase PTPN23 for example leads to a reduced percentage of ciliated cells and to reduced cilia length^25^. In non-ciliated cells it has been demonstrated that PTPN23 regulates endosomal cargo sorting and multivesicular body morphogenesis^40^. Furthermore, the monomeric G-protein Arl13b localizes to primary cilia and mutations of Arl13b lead to defects of sonic hedgehog signaling, which is critically dependent on intact cilia function, while Arl13b also co-localizes with markers of the endocytic recycling pathway like Arf6 and Rab22a and is important for recycling of the membrane protein CD1b^29^,^41^,^42^. Finally, Rab11, a key regulator of recycling, is critical for the accumulation of Rabin8 at the basal body and ciliary activation of Rab8, which is crucial for ciliogenesis^19^,^21^. Thus it is tempting to speculate that SDCCAG3 exerts at least parts of its effects on ciliogenesis by regulating cargo delivery or docking essential for cilia formation. In support of this hypothesis, we have identified a novel interaction of SDCCAG3 with the intraflagellar transport protein IFT88. IFT88 is part of the IFT-B particle, which transports cargo into and within the cilium^6^,^16^. IFT88 mutations are associated with defects in cilia formation and maintenance^30^,^43^. The fact that SDCCAG3 and IFT88 are forming a physical complex suggests that both proteins may act within the same pathway. Our finding that SDCCAG3 interacts with IFT88 opens the possibility that there is a tight relationship between the endocytic pathway and intraflagellar transport in ciliated cells and that this interplay is important for cargo transport to and/or into the cilium. Interestingly, in non-ciliated cells there is already an established relationship between the recycling pathway and components of the intraflagellar transport. Lymphoid and myeloid cells, which lack primary cilia, express proteins for intraflagellar transport but exploit components of this machinery for polarized recycling of the T-cell receptor/CD3 complex to the immune synapse^44^,^45^.

Also in support of the idea that SDCCAG3 might be important for the transport of certain cargo to or into the cilium is our observation that depletion of SDCCAG3 expression levels in IMCD3 cells correlated with impaired localization of the transmembrane protein Polycystin-2 to primary cilia, whereas Rab8 localization to cilia was unaffected. Polycystin-2 is a medically highly relevant cargo as mutations in the Polycystin-2 gene are causing an autosomal dominant form of polycystic kidney disease (ADPKD)^46^. Polycystin-2 is delivered to the cilium directly from the Golgi-apparatus, where vesicles pinch off from the cis-Golgi and are transported to the cilium^47^. Given the subcellular localization of SDCCAG3 to early/recycling endosomes and to the ciliary base, it is unlikely that SDCCAG3 plays a direct role in transporting Polycystin-2 from the Golgi-apparatus to the cilium. However, given its localization at the ciliary base SDCCAG3 could be important for docking of Polycystin-2 containing vesicles. In this context it is interesting that SDCCAG3 has recently been identified in the proximity interactome of several centriole distal appendage proteins like the Outer Dense fiber of Sperm tails-2 (ODF-2) protein or the centrosomal protein 89 (CEP89). The distal centriolar appendages are thought to play an important role in vesicle docking to the ciliary base^39^,^48^,^49^

Polycystin-2 forms a complex with IFT88 and it has been suggested that it may be trafficked as part of a larger protein complex containing IFT88 and other components of IFT like IFT52 and IFT20. Interaction of IFT88 with SDCCAG3 could help with the docking of such vesicles to the ciliary base^34^,^38^. Alternatively, SDCCAG3 could play a role for the transport of Polycystin-2 from the ciliary base into the cilium. This could be either a direct effect of SDCCAG3 or a more indirect effect by delivering other ciliary components important for Polycystin-2 transport into the cilium. Interestingly, the principal importance of recycling pathways for localization of Polycystin-2 to the cilium has recently been demonstrated. Knockdown of phosphoinositide 3-kinase C2alpha, a PI-3 kinase localized at recycling endosomes, interferes with Rab11 activation which subsequently leads to lack of Rab8 localization to cilia. This correlated with a lack of Polycystin-2 localization to cilia, which could be reverted by overexpression of constitutive active Rab8^50^. We have demonstrated that SDCCAG3 does not interfere with Rab8 localization to cilia, however Rab8 is likely not the only component important for ciliary localization of Polycystin-2 and SDCCAG3 knockdown might interfere with the delivery of such components. Impaired localization of Polycystin-1 and 2 to cilia is a major cause for ADPKD^51^. Thus our finding that SDCCAG3 depletion results in impaired localization of Polycistin-2 to cilia could lead to a new avenue of investigating the trafficking mechanism of Polycystin-2 to cilia. However, further experiments are needed to analyze precisely how SDCCAG3 regulates ciliary localization of Polycystin-2.

## Materials and Methods

### Plasmids and siRNA

Plasmids containing full-length SDCCAG3 have been described earlier^23^. Full-length IFT88 cDNA was isolated from a human kidney cDNA library (Clontech, Saint-Germain-en-Laye, France) and subcloned into pcDNA3 vectors (Invitrogen, Darmstadt, Germany) containing an N-terminal myc- or EGFP-tag. SDCCAG3 GST-fusion proteins were generated by inserting the indicated sequence fragment into pGEX-6p-1 vectors (GE Healthcare, Munich, Germany).

Allstar negative control siRNA (cat no. 1027280), SDCCAG3 no.1 (cat no.SI04185888) and no.3 siRNA (cat no. SI00713013), mSDCCAG3 siRNA (Cat no. SI04945885) were purchased from Qiagen (Limburg, Netherlands). SDCCAG3 no.2 siRNA and IFT88 siRNA were synthesized from Dharmacon (Thermo Scientific, Waltham, MA, USA) using following sequences: SDCCAG3, r(AAUUCUAAGCUGAGAAGAAUU);mIFT88, r(AAGGCAUUAGAUACUUAUAAA)dTdT; hIFT88, r(CGACUAAGUGCCAGACUCAUU).

### Cell culture and transfection

HeLa and HEK293 cells were cultured in Dulbecco’s modified Eagle medium (DMEM) supplemented with 10% fetal bovine serum and penicillin/streptomycin. IMCD3 and RPE1 cells (both American type Culture Collection, ATCC) were cultured in DMEM/F12 supplemented with 10% fetal bovine serum, 0.3% sodium bicarbonate and penicillin/streptomycin. RPE1 cells stably expressing Arl13b-GFP were cultured in DMEM/F12 full medium containing 400 μg/ml of G418. For cilia formation induction, RPE1 cells are shifted to DMEM/F12 medium containing 0.2% fetal bovine serum for 48 h, and IMCD3 cells were cultured overnight in DMEM/F12 medium containing no fetal bovine serum.

For DNA transfection, cells were transfected with Polyfect (Qiagen) or Lipofectamine 2000 (Invitrogen) according to the manufacturer’s protocol. siRNA transfections in RPE1 and IMCD3 cells were carried out by using Lipofectamine 2000.

### Antibodies

For western-blot, the following antibodies were used (applied dilution is indicated): rabbit anti-SDCCAG3 (Ab2) (Sigma, HPA029303, dilution 1:500), rabbit anti-IFT88 (Proteintech, 13967-1-AP, dilution 1:800), mouse anti-EEA1 (BD Transduction Lab., Clone 14; dilution 1:100). mouse anti-ß-tubulin (Sigma, T8328, dilution1:5000), rabbit anti-GFP (Invitrogen, A11122, dilution 1:2000), mouse anti-myc (Gentaur, 04-CMYC-9E10, dilution 1:2000). Antibodies directed against the following proteins were used in immunofluorescence: rabbit anti-SDCCAG3 (Ab2) (Sigma, antigenic region aa263-411, HPA029303, dilution 1:100), rabbit anti-SDCCAG3 (Ab1) (Proteintech, 15969-1-AP, antigenic region aa188-412, dilution 1:300), rabbit anti-IFT88 (Proteintech, 13967-1-AP, dilution 1:300), mouse anti-acetylated tubulin (Sigma, T7451, dilution 1:300), rabbit anti-acetylated tubulin (Cell signalling, #5335, 1:800), mouse anti-pericentrin (Abcam, ab4448, dilution 1:300), mouse anti-polycystin 2 (Santa Cruz, sc-28331, diltion 1:300), and mouse anti-rab8 (Proteintech, 55296-1-AP dilution 1:200).

### Western blot

Cells were lysed in 1 × PBS buffer (137mM NaCl, 2.7mM KCL, 10mM Na_2_HPO_4_, 1.8mM KH_2_PO_4_) supplement with 0.5% Triton X-100 and 1 × protease inhibitor (complete EDTA-free protease inhibitor cocktail, Roche). Lysates were collected by centrifugation at 16000 g, 4 °C for 10 min and the protein concentration was measured by DC^TM^ protein assay kit (Bio-Rad Laboratories, Hercules, California, USA). Proteins were separated by SDS-PAGE gel electrophoresis and then transferred onto nitrocellulose membrane. After blocking with 5% not-fat milk for 1 h, membranes were incubated with the relevant antibodies and detected by Pierce^TM^ ECL Western Blotting Substrate (Life Technologies, Carlsbad, CA, USA). Alternatively, blots were detected using the LI-COR Odyssey Sa imaging system (LI-COR Biosciences, UK).

### Protein expression and purification

GST-fusion proteins were expressed in E. coli BL21 (DE3) for 4 h at 18 °C and then lysed in 1 × PBS, 0.5% Triton X-100 via ultrasound sonication (MSE Ltd, London, UK). Lysates were cleared by centrifugation at 4000 g, 4 °C for 20 min. Purification of proteins was performed using glutathione agarose 4B (Macherey Nagel, Dueren,Germany) according to manufacturer’s protocol. In brief, lysates were incubated with 400 μl glutathione agarose 4B beads which were prewashed three times with 1 × PBS, 0.5% Triton X-100 for 4 h at 4 °C. The beads were collected by centrifugation at 3000g for 4 min, and then were washed with 1 × PBS, 0.5% Triton X-100 for three times. In the end, the beads were suspended in 400 μl 1 × PBS, 0.5% Triton X-100 with protease inhibitor to make a 50% slurry solution and store at −20 °C.

### Pulldown experiment

For GST pulldown, cells were lysed in 1 × PBS, 0.5% Triton X-100. Equal amounts of GST-fusion proteins bound to glutathione agarose 4B beads were added to lysates of HeLa or Cos7 cells transfected with the indicated expression constructs for 48 h. Lysates and beads were incubated for 3 h at 4 °C and washed three times with 1 × PBS, 0.5% Triton X-100. Bound proteins were eluted in 2 × Laemmli buffer at 95 °C and then subjected to Western-blotting.

For GFP-Trap pulldown experiments, cells were lysed in lysis buffer (50mM HEPES, pH 7.5, 150mM NaCl, 1.5mM MgCl_2_, 1mM EDTA, 10% glycerol, 1% Triton X-100) and incubated for 2 h with GFP-Trap^®^_A (ChromoTek, Planegg-Martinsried, Germany) at 4 °C. Subsequently, the beads were washed with washing buffer (50mM HEPES, 10% glycerol, 50mM NaCl, 0.1% Triton) three times and elution steps were carried out as described above.

### Immunofluorescence

Cells grown on coverslips were fixed in 4% paraformaldehyde PFA for 20 min and permeablized with 0.5% Triton-X100. After blocking with 2% BSA in PBS for 30min, coverslips were incubated with indicated primary antibodies for 1 h at room temperature in 2% BSA in PBS. Following three times washing with PBS, cells were incubated with Alexa-488-, Alexa-594- or Alex-633-conjugated secondary antibodies for 1 h. Cells were then washed three times with PBS and mounted with VECTASHIELD mounting medium (Vector Laboratories, Burlingame, CA, USA). Images were acquired by Olympus Epifluorescence microscopy, Perkin Elmer Ultraview VoX spinning disc confocal or Nikon A1 confocal microscopy.

### Cell cycle analysis

For cell cycle analysis, 24 h after siRNA transfection, RPE1 cells were serum starved with 0.5% FBS containing medium. 48 h later, the cells were detached and fixed in ethanol for 30 min at room temperature, followed by a wash with PBS for 2 times. After incubating with 400ul propidium iodide staining solution (100 μg/ml RNAse A, 50 μg/ml propidium iodide) for 30 min at room temperature, the cells were analyzed for DNA content by fluorescence activated cell sorting (FACS) on a BD™ LSR II flow cytometer.

### Statistical analysis

Statistical analysis was performed using paired two-tailed Student’s t-test. For cilia length, graphs were drawn using GraphPad Prism software (GraphPad Software) and an F-test was performed to test the variance between the groups.

## Additional Information

**How to cite this article**: Yu, F. *et al*. The serologically defined colon cancer antigen-3 (SDCCAG3) is involved in the regulation of ciliogenesis. *Sci. Rep.* **6**, 35399; doi: 10.1038/srep35399 (2016).

## Supplementary Material

Supplementary Information
