# Supplementary material for: The serologically defined colon cancer antigen-3 (SDCCAG3) is involved in the regulation of ciliogenesis
Source: Sci Rep. 2016 Oct 21;6:35399. doi: 10.1038/srep35399 (PMC5073310; doi:10.1038/srep35399)
Supplement: Supplementary Information [file srep35399-s1.pdf]

Supplementary information

Title: The serologically defined colon cancer antigen-3 (SDCCAG3) is involved in the regulation of ciliogenesis

Fangyan Yu[1,2,3], Shruti Sharma[1,3], Agnieszka Skowronek[1], and Kai Sven Erdmann[1,*]

[1]Department of Biomedical Science & Centre of Membrane Interactions and Dynamics, University of Sheffield, S10 2TN Sheffield, UK

[2] current address: Harvard Center for Polycystic Kidney Disease Research and Renal Division, Department of Medicine, Brigham and Women's Hospital, Harvard Medical School, Boston, MA 02115, USA.

[3]Both authors contributed equally to this study

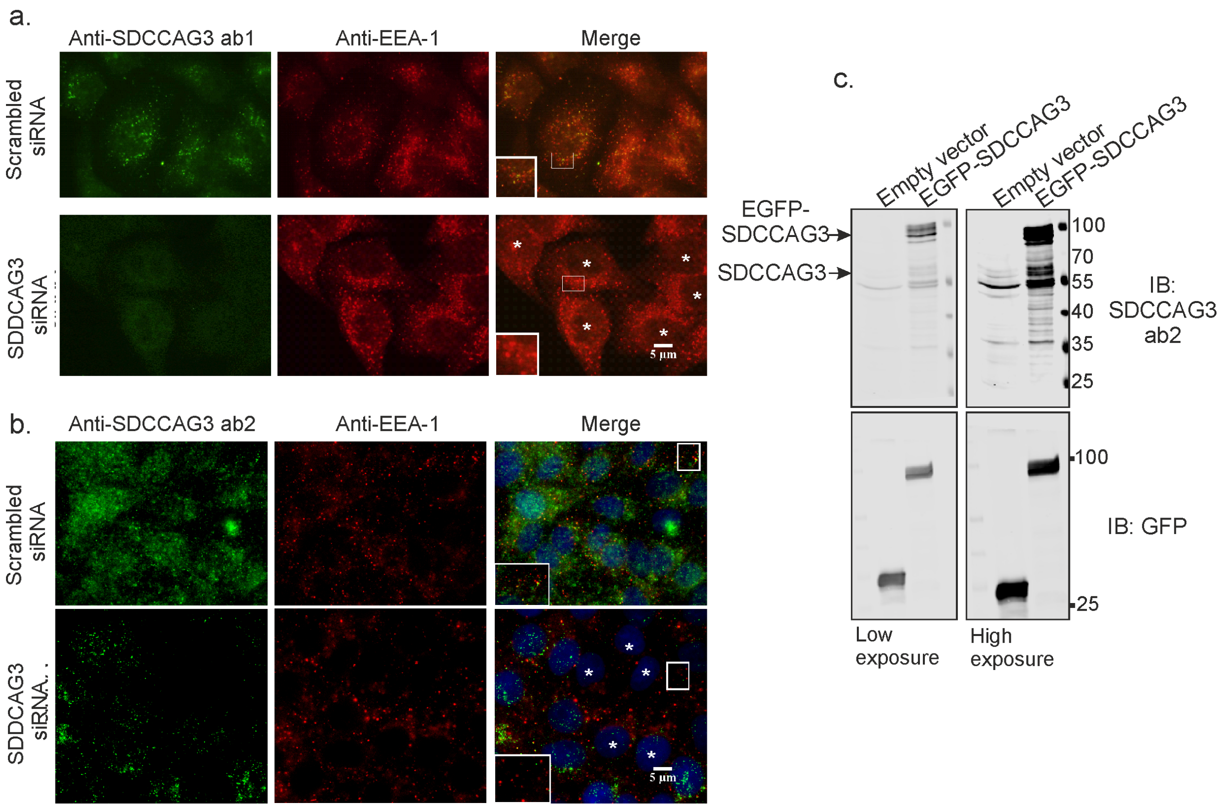

Supplementary figure S1 Specificity test of the anti-SDCCAG3 antibodies (ab1 and ab2). HeLa cells were treated with SDCCAG3 no.2 or scrambled siRNA and analysed by immunofluorescence analysis using SDCCAG3 antibody ab1 (a) or SDCCAG3 antibody ab2 (b). Asterisks represent cells that have none or very minimal staining for SDCCAG3 after knockdown. Scale bar represents 5μm. (c) Immunoblot analysis of HeLa cells (total lysate) transfected with expression vectors for EGFP (empty EGFP vector) or for EGFP-SDCCAG3 and immunoblotted using antibody SDCCAG3 ab2 (upper panel) or anti-GFP antibody (lower panel). Note antibody ab2 efficiently recognizes EGFP-SDCCAG3 but not EGFP alone. The faint bands (in the empty vector lane) at higher exposure marked with SDCCAG3 (arrow) represent endogenous SDCCAG3.

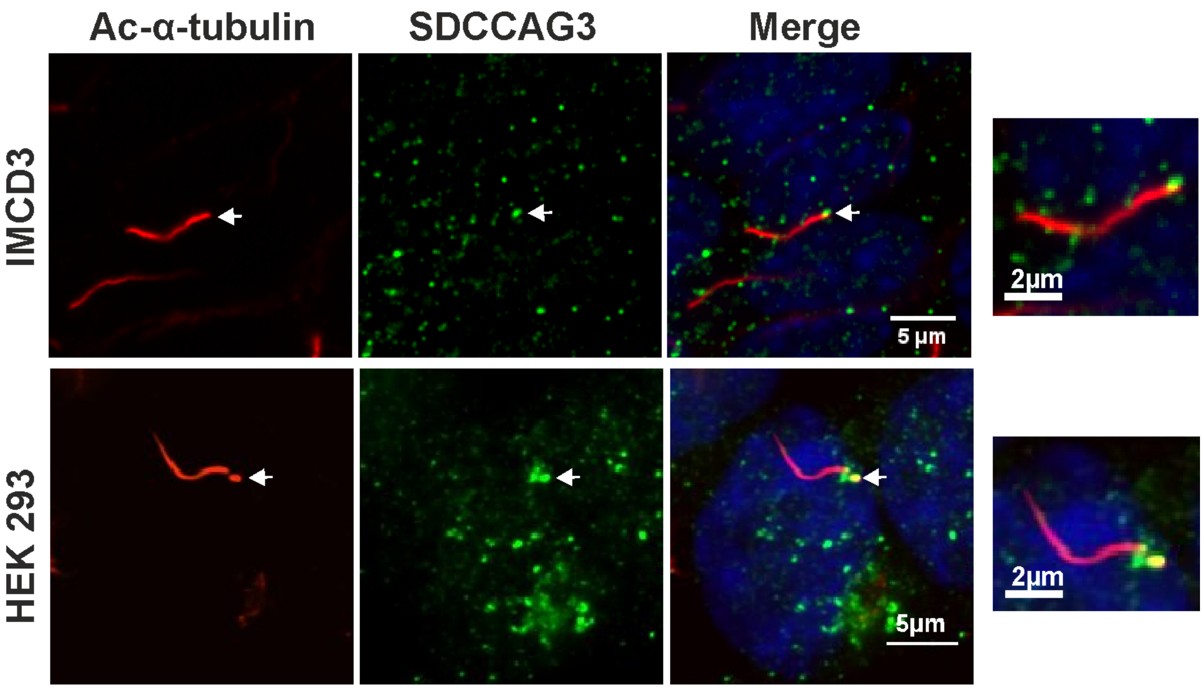

Supplementary figure S2 SDCCAG3 localizes to primary cilia. IMCD3 and HEK293 cells were serum starved for 48 h, 16 h and 24 h respectively to induce cilia. The cells were then fixed and immunostained for SDCCAG3 (*Proteintech-antibody)* and acetylated-α-tubulin. Scale bar represents 5 μm. Inset, scale bar represents 2 μm.

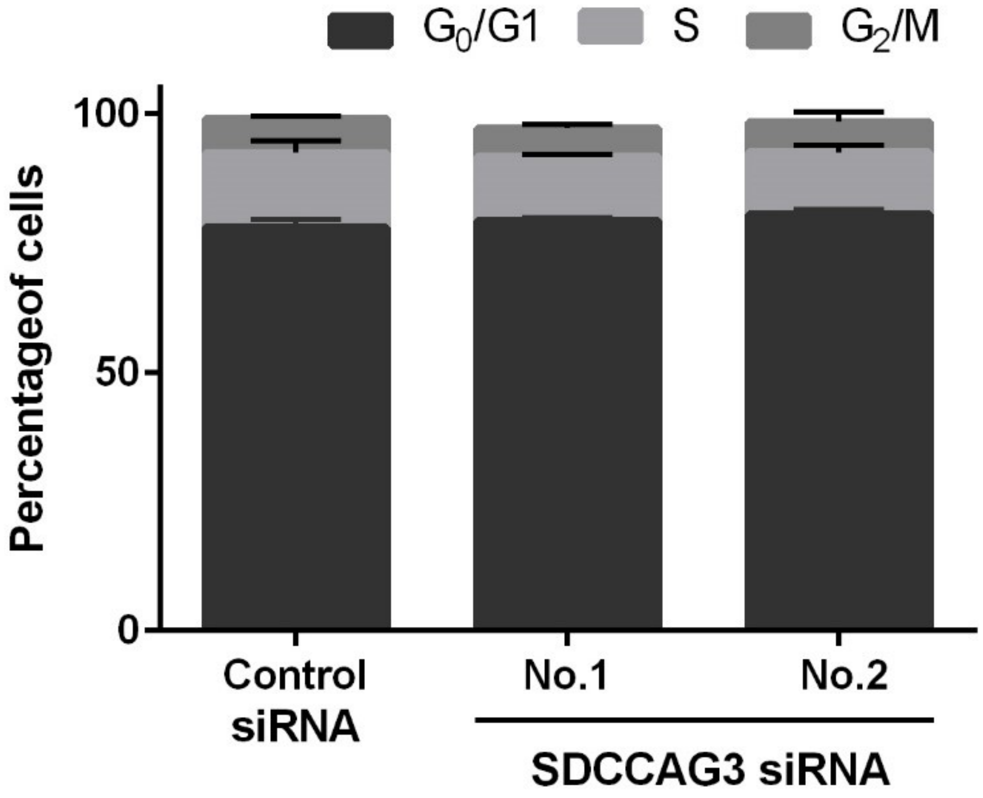

Supplementary figure S3 SDCCAG3 depletion does not affect cell cycle in serum-starved RPE1 cells. RPE1 cells were transfected with control or SDCCAG3 siRNA for 24 hours and were then cultured in serum-free medium for additional 48 hours. Cells were fixed with 70% cold ethanol, stained with propidium iodide, and analyzed by flow cytometry.

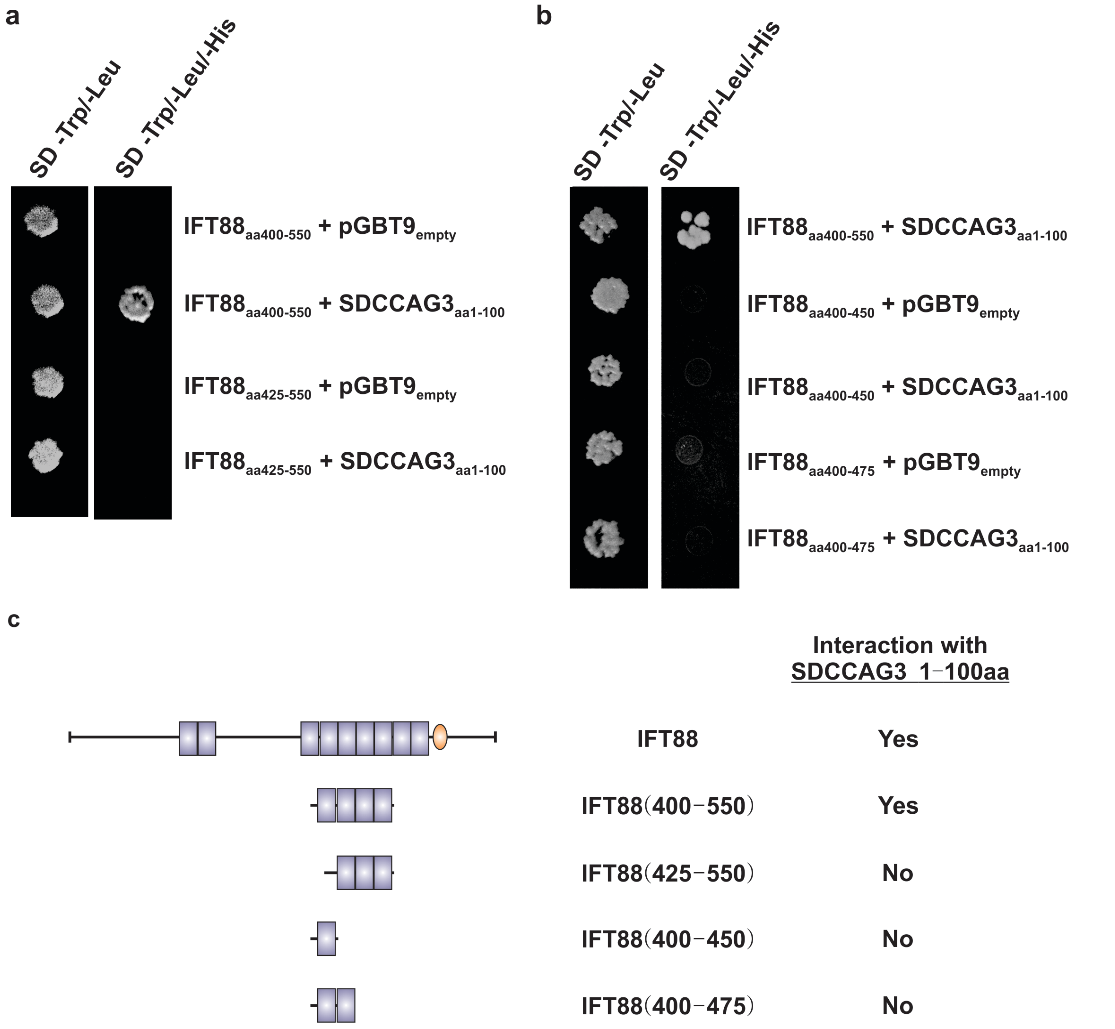

Supplementary figure S4 Mapping of IFT88 binding site in yeast. **(a)** Test of the interaction of IFT88 aa400-550 and IFT88 aa425-550 with SDCCAG3. Expression plasmids for SDCCAG3 aa1-100 or the empty plasmid (pGBT9) were co-transfected with plasmids expressing IFT88 aa400-550 or IFT88 aa425-550 into yeast. The interaction was tested on minimal media plates. Growth on media deficient for tryptophan (Trp), leucine (Leu) and histidine (His) indicates interaction of the corresponding proteins. **(b)** Test of the interaction of IFT88 400-450 aa and IFT88 400-475 aa with SDCCAG3. **(c)** Schematic diagram of the mapping strategy.

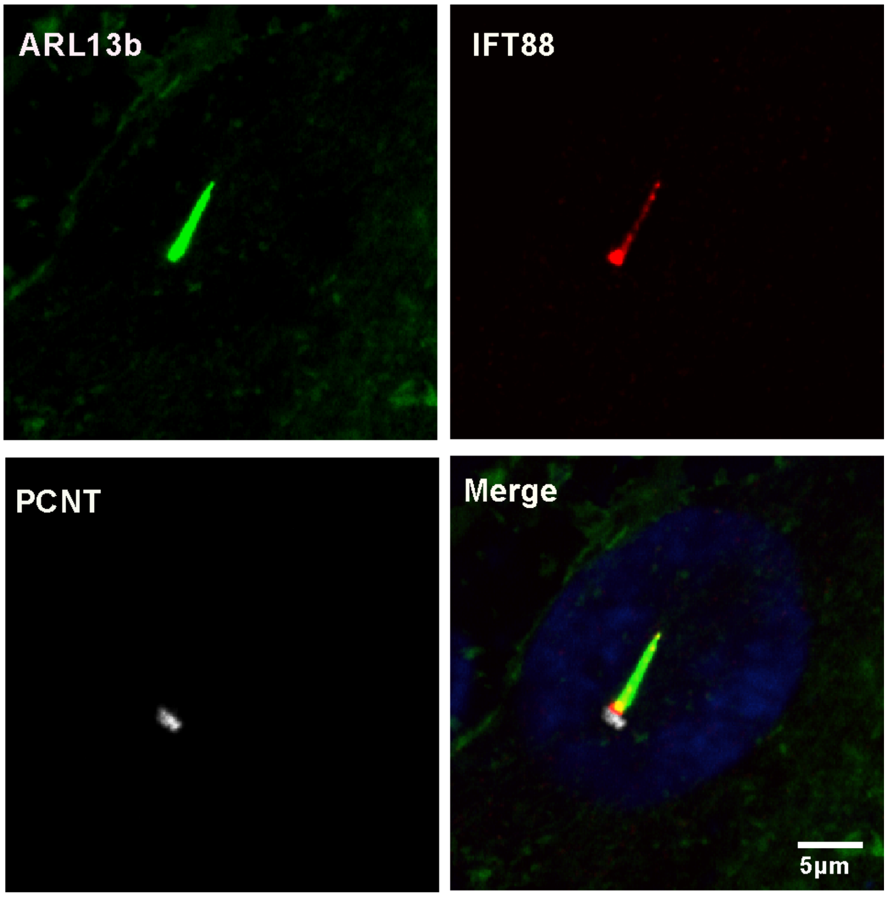

Supplementary figure S5 Localization of IFT88 in Arl13b-RPE1 cells. RPE1 stably expressing ARL13b were serum starved for 48 h and stained with anti-IFT88 antibody, anti-pericentrin antibody and DAPI. Scale bar represents 5 μm.

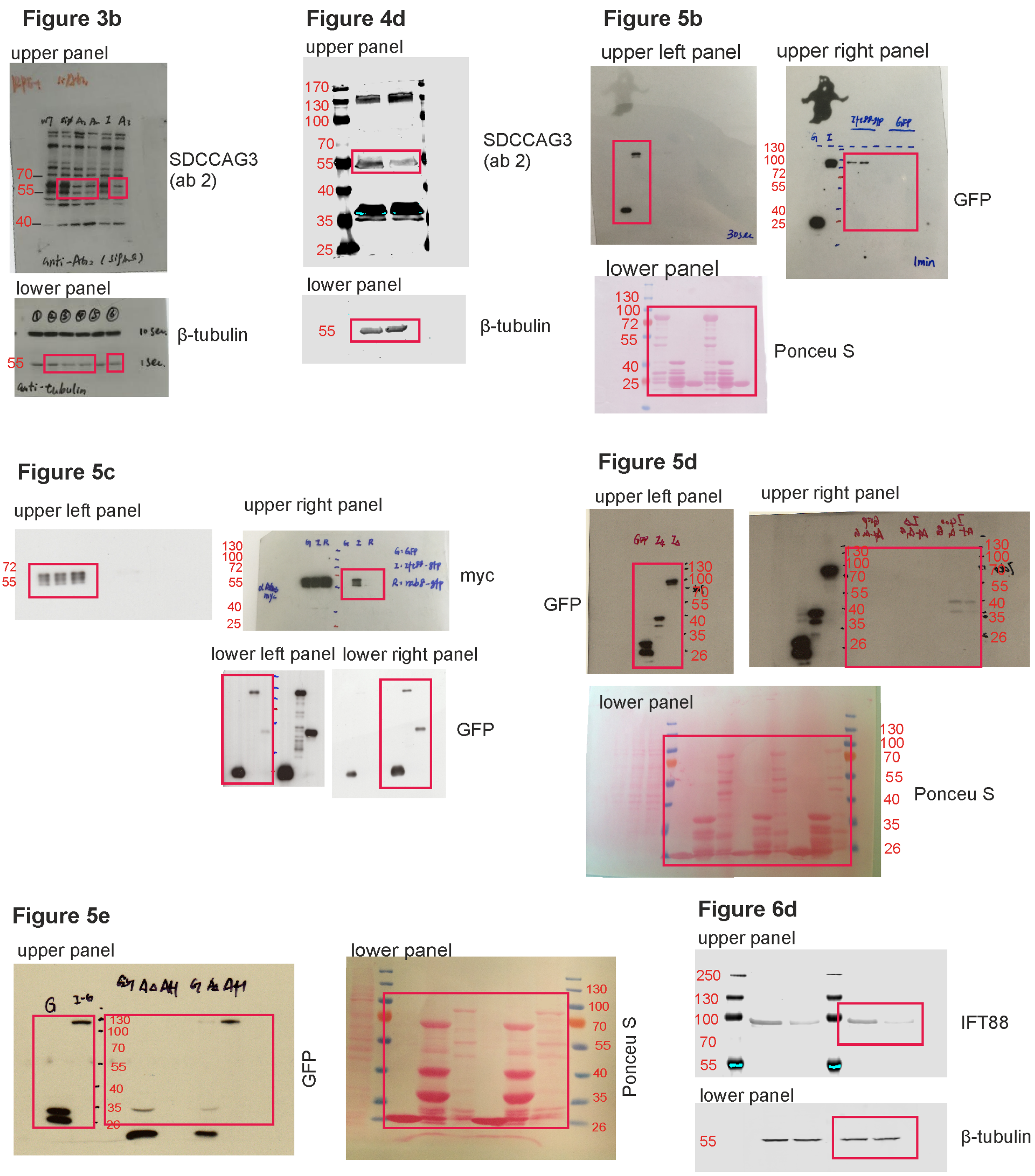

Supplementary figure S6 Full-blots used to prepare figures 3, 4, 5, and 6. Shown are scans from films or Ponceau stained nitrocellulose for figures 3 and 5. For figures 4 and 6 blots were imaged with the LI-COR Odyssey Sa. system.
